# Motor learning in multijoint virtual arm movements with novel kinematics

**DOI:** 10.1038/s41598-024-60844-7

**Published:** 2024-05-07

**Authors:** Nagisa Inubashiri, Shota Hagio, Motoki Kouzaki

**Affiliations:** 1 Laboratory of Neurophysiology, Graduate School of Human and Environmental Studies, Kyoto University, Kyoto, Japan; 2 Laboratory of Motor Control and Learning, Graduate School of Human and Environmental Studies, Kyoto University, Yoshida-nihonmatsu-cho, Sakyo-ku, Kyoto, 606-8501 Japan; 3 Unit of Synergetic Studies for Space, Kyoto University, Kyoto, Japan

**Keywords:** Motor control, Learning and memory

## Abstract

Humans move their hands toward precise positions, a skill supported by the coordination of multiple joint movements, even in the presence of inherent redundancy. However, it remains unclear how the central nervous system learns the relationship between redundant joint movements and hand positions when starting from scratch. To address this question, a virtual-arm reaching task was performed in which participants were required to move a cursor corresponding to the hand of a virtual arm to a target. The joint angles of the virtual arm were determined by the heights of the participants’ fingers. The results demonstrated that the participants moved the cursor to the target straighter and faster in the late phase than they did in the initial phase of learning. This improvement was accompanied by a reduction in the amount of angular changes in the virtual limb joint, predominantly characterized by an increased reliance on the virtual shoulder joint as opposed to the virtual wrist joint. These findings suggest that the central nervous system selects a combination of multijoint movements that minimize motor effort while learning novel upper-limb kinematics.

## Introduction

Humans control movements, precisely managing hand positions or tool endpoints. These endpoint movements rely on how the central nervous system (CNS) coordinates multiple joint movements with redundancy. Understanding this coordination is fundamental for human motor control^1^. The CNS employs internal models, which are neural representations linking joint movements to the corresponding movements of the hand or tool endpoints^2–4^. The development of internal models is essential for learning movements involving novel kinematics; these developments have been observed in infant motor development and in adapting to prosthetic limbs^5,6^. However, a key question remains as to how the CNS orchestrates multiple joint movements while learning internal models for novel body kinematics, particularly when starting from scratch.

The CNS controls multiple joint movements considering kinematic properties such as the distance between the joint and the hand. Previous studies have investigated the learning of multiple joint movements, focusing on the developmental change in reaching movements in infants^7–10^. Berthier et al. showed that infants largely use their shoulder to move their hands to an object^8^. This finding is believed to result from differences in the rate of maturation of the neuromuscular system between the proximal and distal parts of the body. For this reason, it is uncertain whether adults use joints in a similar way when learning new internal models of kinematics. The CNS may modularly control the degrees of freedom of the body as a control strategy to overcome redundancy in the motor system^11–13^. In contrast, it has been suggested that the covariation pattern of the degrees of freedom can be reproduced by the biomechanical properties of the musculoskeletal system^14^. In addition, because of the dynamic interactions and the anatomical connections by biarticular muscles between segments^15,16^, each joint movement is not completely independent. Accordingly, it has not been possible to isolate whether the observed joint movement patterns are due to control strategies or simply biomechanical constraints. Elucidation of the control strategy for multijoint movements focusing on each joint, which has different kinematic properties, leads to an understanding of the learning process of joint control patterns. Hence, an experiment that minimizes the effects of biomechanical constraints should be conducted to understand the control strategy employed by the CNS to control multiple joint movements during the motor learning process.

Learning internal models of novel body kinematics from scratch is considered to be a different motor learning type than motor adaptation, which has received much attention in the field of motor control^17,18^. Previous studies have shown that prior motor and/or sports experience influences future motor learning^6,19,20^. Additionally, the motor learning process may be affected by individual differences in the kinematics to be learned, such as limb length and muscle properties. Thus, it is necessary to reduce the effect of prior motor experience and body size when investigating learning internal models of novel body kinematics. Rohde et al. constructed a new experimental task on the learning of internal models of kinematics that satisfied this demand^21^. In this previous study, participants had to learn a sensorimotor mapping that reflected the kinematic properties of a three-joint virtual arm. This task is effective for investigating strategies used by the CNS to control multiple joint movements during novel kinematics learning because it reduces biomechanical constraints such as limb length and biarticular muscles. Although their study showed that the low-dimensional structures of joint movements are formed gradually, how control of each joint movement is modulated during novel body kinematics learning is unclear. Therefore, we investigated changes in multiple joint movements of the virtual arm as the internal model of the virtual arm was learned.

The purpose of this study was to examine how the CNS controls multiple joint movements while forming new neural representations of limb kinematics. To this end, we adopted the virtual-arm reaching task^21^ and measured the ability of endpoint control to quantify the learning of new limb kinematics. We also examined changes in the use of virtual arm joints, and then, assessed the associations between endpoint control ability and joint movement control characteristics.

## Results

**Figure 1:**
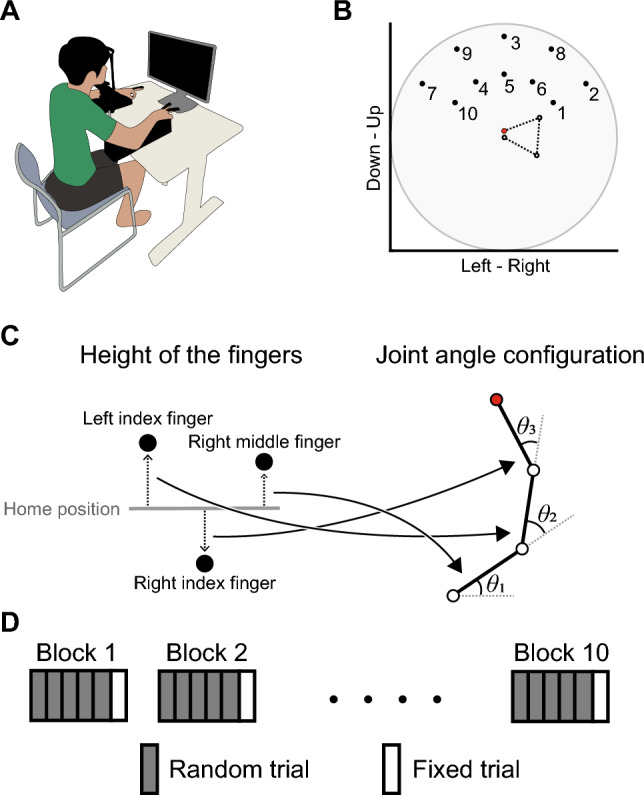
Experimental setup. (**A**) Illustration of the experimental setup. Participants sat in a stable chair with their chin resting on a chinrest and their arms resting on armrests on a table. The direct vision of their fingers was occluded by wearing glasses with plates. (**B**) The initial posture of the virtual arm (dotted line and block circles) and the 10 target positions (black dots) in the task space. The numbers near the targets indicate the ordering of the targets in the fixed trial. The gray area represents reachable space. The endpoint of the virtual arm was shown as the red cursor on the monitor. The posture of the virtual arm was invisible during the experiment. The initial angles (in radians) of the shoulder, elbow, and wrist joints were 0.5, 2, and 2, respectively. Ten targets were distributed in a one-third segment of the task space. (**C**) The participants’ finger positions were mapped to the cursor position. The vertical positions of the left index, the right index, and the right middle fingers were linearly transformed to joint angles of the virtual arm. The assignment of fingers to joints and of flexion/extension between fingers and joint movements was randomized for each participant. The endpoint (red circle) of the virtual arm was displayed as the cursor. (**D**) Protocol of this experiment. The experiment consisted of ten blocks of six trials. During a trial, ten targets were presented either in random order (random trial) or in a fixed order (fixed trial). In each block, five random trials were followed by a fixed trial.

Participants performed a 3-joint virtual-arm reaching task in which they moved their fingers (left index, right index, and right middle fingers) in the vertical direction to manipulate the endpoint of the virtual arm (Fig. 1). The heights of their fingers were converted into joint angles (shoulder, elbow, and wrist joint) of the virtual arm, and the endpoint of the virtual arm was displayed as a cursor on the monitor. In order to investigate whether the stereotyped control strategy for the virtual arm would be observed regardless of the finger-to-joint mapping, the assignment of fingers to joints and of flexion/extension between fingers and joint movements was randomized across participants. In the task, participants were asked to reach the cursor to a target as fast and straight as possible. In each trial, 10 targets spread across the task space were displayed in sequence. Ten targets were presented either in random order (random trial) or in fixed order (fixed trial). The experiment consisted of ten blocks, each consisting of six trials (5 random trials followed by the fixed trial).

### Task performance

**Figure 2:**
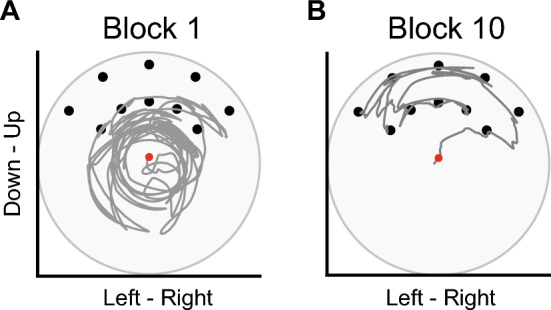
The cursor trajectories of the representative participant. Cursor trajectories during the fixed trial in the first and last blocks for the same participant. (**A**) The first block. (**B**) The last block. The gray line indicates the cursor trajectory. The black and red dots represent the target position and the initial position of the cursor, respectively. The gray area represents reachable space.

**Figure 3:**
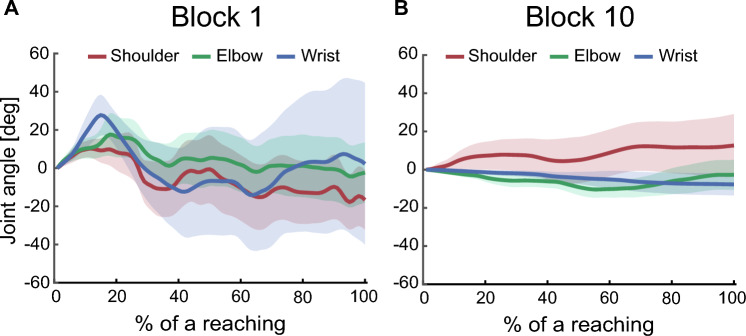
The joint angles of the representative participant. The joint angles of the virtual arm of the same participant during a single point-to-point reaching. (**A**) The fixed trial in the first block. (**B**) The fixed trial in the last block. Angles are expressed relative to the initial joint configuration. The red, green, and blue lines indicate the average over 10 point-to-point reaching movements of the virtual shoulder, virtual elbow, and virtual wrist joints, respectively. The shaded area represents the standard error of the mean (SEM).

**Figure 4:**
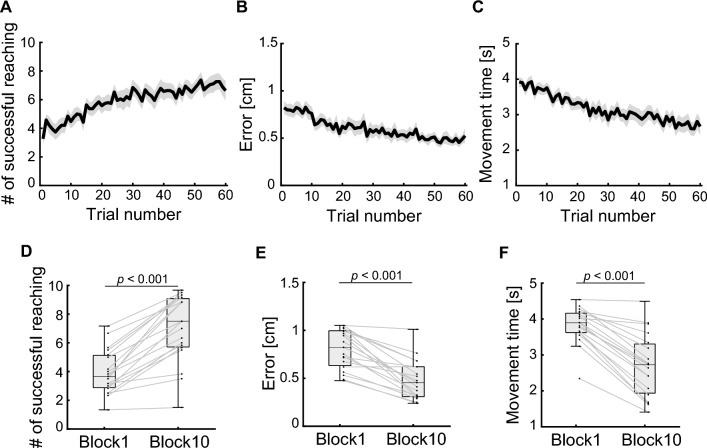
Task performance. (**A**) The number of successful reaching movements. (**B**) The directional error of the cursor. (**C**) The movement time. The black line indicates the average across participants. The shaded area represents the SEM. (**D–F**) Boxplot in the first (trial number: 1–6) and last (trial number: 55–60) block of task performance. Each dot represents the mean value during each block of an individual participant.

Figure 2 shows the cursor trajectories of a representative participant in the first and the last trials of the fixed trials. The cursor trajectory was curved remarkably and reached only targets close to the start position of the cursor in the first trial (Fig. 2A), whereas it was approximately straight and reached all targets in the last trial (Fig. 2B). To visualize typical examples of virtual joint movements during point-to-point goal-directed movements, the joint angles from movement onset on a current target to the next target appearance in a trial were extracted, i.e., ten reaching movements were extracted in a trial, and the angle at movement onset was subtracted from the joint angles. These joint angle data were then time-normalized to 100 points and averaged at each time point. Figure 3 shows these joint angles for all joints of the virtual arm of a representative participant in the first and the last trials of the fixed trials. In the first block, all virtual joints were used, and the amount of joint angular changes was large (Fig. 3A). On the other hand, the virtual shoulder was mainly used, and the amount of angular change in all joints decreased in the last block (Fig. 3B). Figure 4A shows the mean number of successful reaching movements in a trial. The mean number of successful reaching movements increased gradually during the experiment. The mean number of successful reaching movements in the last block was significantly higher than that in the first block (the first block, 3.96 ± 1.55; the last block, 7.04 ± 2.34; *p* < 0.001). Figure 4B shows the mean directional errors between the cursor and the straight-line path between targets during the experiment. This value also decreased gradually during the experiment. The mean directional error in the last block was significantly smaller than that in the first block (the first block, 0.81 ± 0.20 cm; the last block, 0.48 ± 0.21 cm; *p* < 0.001). In addition, Fig. 4C shows the mean movement times during the experiment. The mean movement time in the last block was significantly shorter than that in the first block (the first block, 3.82 ± 0.49 s; the last block, 2.70 ± 0.87 s; *p* < 0.001).

### The amount of joint angular change

**Figure 5:**
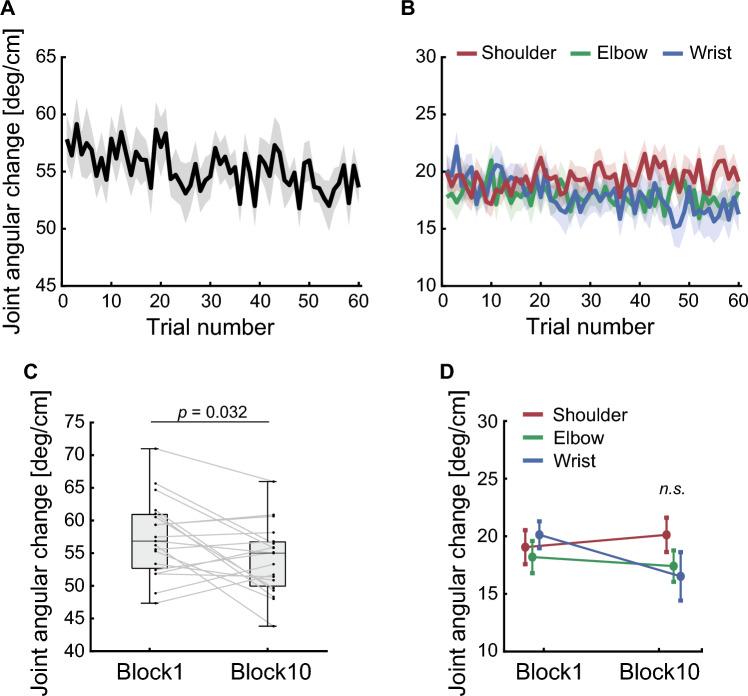
The amount of joint angular change. The amount of joint angular change summed over all joints of the virtual arm. (**A**) The black line indicates the average across participants. (**B**) The amount of joint angular changes in the virtual shoulder, the virtual elbow, and the virtual wrist joints averaged across participants. The red, green, and blue lines indicate the shoulder, the elbow, and the wrist joints, respectively. The shaded area represents the SEM. (**C**) Boxplot in the first (trial number: 1–6) and last (trial number: 55–60) block of joint angular change. Each dot represents the mean value during each block of an individual participant. (**D**) Joint angular change for each joint in the first (trial number: 1–6) and last (trial number: 55–60) block. All error bars represent the SEM.

To evaluate the ability to move the cursor, we quantified the amount of joint angular changes in the virtual arm, normalized by displacements of the cursor during a trial. Figure 5A shows the time course of the mean normalized joint angular change summed over all joints. The normalized joint angular change was significantly lower in the last block than in the first block (the first block, 57.38 ± 5.96 deg/cm; the last block, 54.05 ± 5.32 deg/cm; *p* = 0.032; Fig. 5C). In contrast, according to the two-way repeated-measures ANOVA, no statistically significant differences were detected for the interaction effect between the joints of the virtual arm and blocks (*F*_2,54_ = 2.86, *p* = 0.066) or for the main effects of the joints of the virtual arm (*F*_2,54_ = 0.98, *p* = 0.382) or blocks (*F*_1,54_ = 1.90, *p* = 0.174) (Fig. 5B,D).

### Relative use of each joint

**Figure 6:**
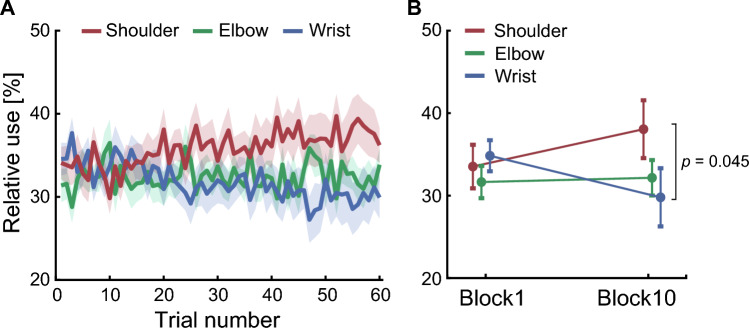
Relative use of each joint. (**A**) Relative use of virtual shoulder, virtual elbow, and virtual wrist joints, averaged across participants. The red, green, and blue lines indicate the shoulder, the elbow, and the wrist joints, respectively. The shaded area represents the SEM. (**B**) Relative use of each joint in the first (trial number: 1–6) and last (trial number: 55–60) block. All error bars represent the SEM.

**Figure 7:**
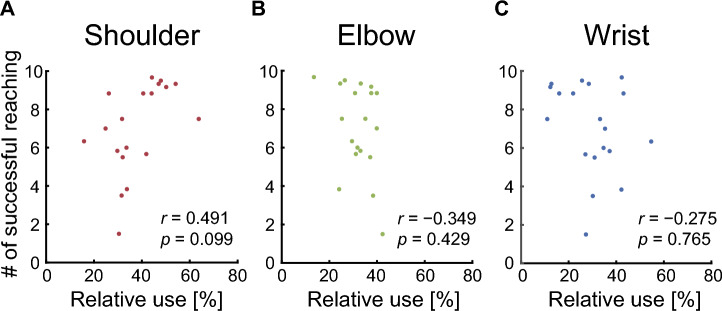
Association between relative use and the number of successful reaching. Associations between relative use of each joint and the number of successful reaching. (**A**) The virtual shoulder joint. (**B**) The virtual elbow joint. (**C**) The virtual wrist joint. The dots indicate individual participants.

To investigate how participants controlled joint movements of the virtual arm, we quantified the ratio of the angular change in each virtual joint to the sum of joint angular changes in all virtual joints. Figure 6 shows the time course of the mean relative use of each joint of the virtual arm. Two-way repeated- measures ANOVA revealed no significant main effect of the joints of the virtual arm (*F*_1,54_ = 1.434 × 10^−10^, *p* = 1.000) or blocks (*F*_2,54_ = 1.75, *p* = 0.184). We observed a significant interaction between the joint and the block (*F*_2,54_ = 3.59, *p* = 0.035). The simple main effects showed a significant difference among joints in the last block (virtual shoulder, 38.1 ± 11.6%; virtual elbow, 32.2 ± 7.1%; virtual wrist, 29.8 ± 11.7%; *p* = 0.049). Post hoc tests showed that the relative use of the virtual shoulder was significantly greater than that of the virtual wrist (*p* = 0.045). In addition, we evaluated the associations between the amount of use of each virtual joint and motor learning ability. As a result, no statistically significant correlation was found between the relative use of joints of the virtual arm and the number of successful reaching movements (shoulder, *r* = 0.491, *p* = 0.099; elbow, *r* =  − 0.349, *p* = 0.429; wrist, *r* =  − 0.275, *p* = 0.765; Fig. 7). Finally, in order to clarify whether the kinematics of the virtual arm were learned regardless of the finger-to-joint mapping, we calculated the relative use of fingers (the left index, right index, and right middle fingers). Two-way repeated-measures ANOVA showed that no significant differences were detected for the interaction effect between the fingers and blocks (*F*_2,54_ = 0.475, *p* = 0.636) or for the main effects of the fingers (*F*_2,54_ = 0.204, *p* = 0.816) or blocks (*F*_1,54_ = 1.287 × 10^−10^, *p* = 1.000) (see Supplementary Fig. S2 online).

## Discussion

In this study, we aimed to investigate how the CNS controls multiple joint movements while learning new limb kinematics. To this end, we used the virtual-arm reaching task, in which each finger movement of the participants corresponded to each joint movement of the virtual arm. Participants had to learn the relationship between their finger movements and the position of the cursor indicating the endpoint of the virtual arm to successfully perform the task. Participants were able to move the cursor to the target straighter and faster after learning than in the initial phase of learning (Fig. 4). As the experiment progressed, participants were able to move the cursor with fewer joint movements of the virtual arm after learning (Fig. 5). After learning, the shoulder joint of the virtual arm was used more than the wrist joint was (Fig. 6).

In the current study, we adopted the reaching task, in which participants manipulate the virtual arm^21^. Since this task minimizes the influence of biomechanical constraints and previous motor experience on joint control, we were able to explore strategies for controlling multiple joints while learning novel kinematics. Participants in the current study were not told that the cursor movement depended on the posture of the virtual arm. Furthermore, there were many possible combinations of multiple joint movements to complete this task. Even in this situation, the common joint control characteristic of greater use of the shoulder joint of the virtual arm than of the wrist joint was observed after learning. The results indicate that participants learned the relationships between the joint angles and the endpoint of the virtual arm behind the acquisition of finger-to-cursor mapping.

The primary outcome of this study was greater relative use of the shoulder joint than of the wrist joint in the virtual arm (Fig. 6). This finding may be attributed to the different effectiveness of virtual arm joints in manipulating the cursor. It has been known that the multijoint movements are determined by taking into account the effect of each joint on the endpoint variability^22^. The virtual shoulder joint, which is the farthest from the endpoint of the virtual arm, translates finger movements into larger cursor movements than the other virtual joints. Consequently, virtual shoulder joint movements were effective at moving the cursor over a wide area of the task space. This preference for the proximal joint of the virtual arm is consistent with what has been observed in the Goal Babbling simulation, which is a computational approach to novel internal model learning in robotics^21^. In this computational approach, more effective joints were actively used when learning new inverse kinematics, which leads to minimizing effort costs when exploring motor solutions^23,24^. In contrast, human participants tended to use ineffective joints the most during learning^21^. This opposite trend between the Goal Babbling simulation and human behavior was interpreted to be due to the human preference for using joints that were less affected by motor noise. Although in this previous study, participants saw only the ellipse, which reflected the distance between the endpoint of the virtual arm and the target location, we provided visual feedback about the cursor location. Visual feedback about the cursor in the task space might have enabled active use of the proximal joint of the virtual arm. Furthermore, the training volume of the current study was greater than that of the previous study. Because motor skill learning is considered a form of motor learning that takes a long time^25^, extensive training experience improves the ability of the virtual arm to learn kinematics. Qualitatively, absolute virtual wrist joint use decreased as the experiment progressed, whereas shoulder use did not change (Fig. 5B). The extensive training may have led to a greater reliance on proximal joints over distal joints, allowing participants to move the cursor with fewer finger movements. Taken together, the greater use of the virtual shoulder joint relative to the virtual wrist joint with the improvement in cursor control indicates that the CNS has learned joint control for the virtual arm to move the cursor effortlessly. In addition, the greater use of the virtual shoulder joint is consistent with the joint control characteristics during the reaching of young infants^8^. The predominant use of the proximal joint in infants is believed to be due to the neural development of the proximal body parts after birth^26,27^. Our results suggest that the use of the proximal joint relative to the distal joint is explained by the neural strategy, which reaches with fewer joint movements, as well as the neural development.

Additionally, to estimate the optimal usage of the virtual joints, we simulated the generation of desired trajectories for the virtual arm. This simulation assumed that the endpoint of the virtual arm follows the shortest straight-line path between all pairs of targets, while minimizing the angular changes of the virtual joints (see Supplementary Methods online). Consequently, the relative use of the virtual joints from the simulated data was found to be higher in the middle joint (see Supplementary Fig. S1 online), which contrasts with our experimental results indicating a higher relative use of the virtual shoulder joint. This discrepancy was thought to be due to differences in learning stages. We simulated the optimal control for the virtual arm, assuming the participants learned well. Motor skill learning is a form of learning that takes a long time^25^. Thus, the ability of virtual arm control improved, but participants were still considered to be in the early stages of learning. Therefore, it is likely that the participants in our study prioritized moving the cursor on the screen across a large area while minimizing motor effort costs rather than moving the cursor in a straight line.

There were individual differences in cursor control ability after learning. We found no statistically significant correlation between the relative use of each joint of the virtual arm and the number of successful reaching movements (Fig. 7). However, there was a trend that the relative use of the virtual shoulder was positively correlated with the motor learning performance. A potential explanation for this trend is that strategies to control joints during motor exploration affect motor learning performance. Previous studies have demonstrated that motor exploration may be a key factor in acquiring new internal models^28–31^. Similarly, Berger et al. reported that long-time exploration of task space contributes to the learning of new muscle coordination patterns^32^. Therefore, the reduced use of the wrist joint of the virtual arm, indicating greater use of the virtual shoulder joint, might have facilitated the exploration of a larger area of task space, which produced improved motor performance.

We also found that the normalized joint angular change summed across all joints decreased as motor learning progressed (Fig. 5A). In the present study, participants moved their fingers to control the cursor, so a decrease in joint angular change indicated a decrease in finger movements. The CNS generates motor commands that minimize motor effort costs when executing well-learned movements^33,34^. It has also been reported that multi-effector movements are coordinated to minimize motor effort costs^35^. In addition, a previous study on skill learning reported that the CNS selects the movement solution that minimizes effort during learning^36^. Similarly, previous studies using arm-reaching tasks have shown that joint configurations at target locations depend on effort^37,38^. Hence, decreasing finger movements suggests that the CNS minimizes motor effort costs while learning new internal models of limb kinematics.

In the task of this study, enslaving, which refers to the involuntary movement of non-intended fingers during finger movements, can occur^39^. Finger enslaving could affect motor learning. To reduce the effect of finger enslaving, we randomized the finger-to-joint mapping across the participants. In addition, we investigated the effect of finger enslaving by examining whether the learning was constrained by the biomechanical structure of the fingers or by the kinematics of the virtual arm. As a result, in the last block, although the relative use was similar among fingers (see Supplementary Fig. S2 online), the relative use of the virtual shoulder was higher than that of the virtual wrist (Fig. 6). This result indicates that the participants learned the kinematics of the virtual arm with little effect of the finger enslavement.

We investigated the neural control strategies employed by the CNS to combine multiple joint movements while learning novel limb kinematics. It should be noted that the task of this study reduced biomechanical constraints such as interaction torque and anatomical connections between joints, and participants also received no proprioceptive information about limb state. Thus, how interactions between biomechanical constraints or proprioceptive information and neural control strategies affect joint control during the learning of new internal models has not been determined. Previous studies have shown that the biomechanical properties of limbs affect neural activity in the primary motor cortex^40^ and joint coordination patterns in redundant motor systems^41^. An experimental task involving the manipulation of a virtual arm with muscles acting on multiple joints or via proprioceptive feedback enables us to understand neural control strategies involving biomechanical constraints or proprioceptive information.

## Conclusion

In this study, we demonstrated that during the learning of redundant multijoint virtual arm movements with novel limb kinematics, the proximal joint was more actively utilized than the distal joint. Furthermore, joint angular displacement decreased as learning progressed. These results suggest that, in the absence of biomechanical constraints such as interaction torque and sensory feedback regarding limb state, the CNS adopts a learning strategy that favors the combination of multiple joint movements while minimizing motor effort costs. These findings enhance our understanding of how the CNS adapts to novel motor tasks, emphasizing the role of joint-specific strategies in motor learning.

## Materials and Methods

### Participants

Nineteen healthy right-handed adults (15 males, 4 females; age: 23.1 ± 2.1 years, mean ± standard deviation [SD]) participated in this study. Participants provided written informed consent to participate in the study prior to the experiment. This study was conducted in accordance with the Declaration of Helsinki, and all procedures were approved by the Local Ethics Committee of the Graduate School of Human and Environmental Studies, Kyoto University (Approval number: 20-H-6).

### Experimental setup

Participants sat in a stable chair with their chin resting on a chinrest and their arms resting on armrests on a table (Fig. 1A). The positions of the chinrest and armrests were adjusted individually for each participant. Participants wore glasses; these glasses occluded the direct vision of their fingers. A 27-inch monitor (refresh rate: 120 Hz) was placed 0.5 m in front of participants at eye level, and the participants were shown a target (0.3 cm radius circle), a cursor (red cursor), a line indicating a home position of their fingers (yellow horizontal line), or a vertical position of their fingers (white cursor). The vertical positions of the fingers were captured by a real-time motion tracking system. Rigid bodies with four or five infrared reflective markers were attached to the left index, right index, and right middle finger. The rigid body coordinates were sampled at 100 Hz by using a three-dimensional optical motion capture system (OptiTrack V100, Natural Point Inc., Oregon, United States) with 4 cameras spaced around the participant’s fingers. NatNet SDK 3.0.1 (https://www.optitrack.jp/support/support01/support01-02/) provided by OptiTrack was used to extract motion data from Motive 2.1.2 software (Natural Point Inc., Oregon, United States, https://www.optitrack.jp/support/support01/). This allowed for rigid body coordinates to be streamed in real time to LabVIEW (National Instruments, Texas, United States). The vertical position of each finger relative to the home position was transformed into a displacement of the joint angle of the virtual arm from the initial joint angle. Moving the finger 1 cm corresponded to a 60-degree change in the joint angle. The assignment of fingers to joint angles of the virtual arm and the correspondence of flexion/extension between fingers and joint movements were randomized among participants.

### Virtual reaching task

Participants performed a virtual reaching task in which they were required to move a cursor corresponding to the endpoint of the virtual arm to a target. (Fig. 1B). This task was redundant because there are many joint configurations of the virtual arm that satisfy the requirements of the task, and this approach allowed us to investigate the process of forming an internal model of novel kinematics from scratch. In the task, participants were asked to reach the cursor to a target on the monitor by moving their three fingers (left index, right index, and right middle finger) in a vertical direction. The cursor and a target were presented on the monitor during the task. The posture of the virtual arm, when participants held all fingers in the home position, was defined as the initial posture q (angles in radians) = (0.5,2,2). The home position of the fingers was defined as the position where all fingers were located at the midpoint of the vertical range of motion (ROM) of each finger. All segments of the virtual arm are of equal length (1 cm), and each joint angle can be varied in the range [$-\pi , \pi$] from the initial angles. The vertical position of each finger relative to the home position was linearly mapped to each joint angle of the virtual arm (Fig. 1C). Joint angles of the virtual arm were expressed as positive for extension and negative for flexion. The 2-dimensional location of the cursor (*x*, *y*) was represented by the following equation:

1 $$\begin{array}{*{20}c} {\left[ {\begin{array}{*{20}c} x \\ y \\ \end{array} } \right] = \left[ {\begin{array}{*{20}c} {L\cos \left( {\theta_{1} } \right) + L\cos \left( {\theta_{1} + \theta_{2} } \right) + L\cos \left( {\theta_{1} + \theta_{2} + \theta_{3} } \right)} \\ {L\sin \left( {\theta_{1} } \right) + L\sin \left( {\theta_{1} + \theta_{2} } \right) + L\sin \left( {\theta_{1} + \theta_{2} + \theta_{3} } \right)} \\ \end{array} } \right]} \\ \end{array}$$

where *L* represents the length of each segment of the virtual arm, with all lengths equal to 1 cm. *θ*_1_,* θ*_2_, and* θ*_3_ denote the joint angles of the shoulder, elbow, and wrist, respectively. As described above, the cursor position (*x*, *y*) corresponded to the endpoint of the virtual arm. Thus, participants were required to change the posture of the virtual arm by controlling the vertical movements of their fingers to move the cursor to the target. Ten targets were evenly placed in one-third of the segment of the circular task space (distances from the initial cursor position: 1.5, 2.5 cm; angles: 30, 60, 90, 120, 150 deg; Fig. 1B).

### Experimental procedure

Before the experiment, we measured the ROM in the vertical direction of each finger while keeping the other fingers immobile to minimize enslaving. As mentioned above, the ROM was used to set the home position of the fingers. During the task, participants were instructed to reach the cursor to the targets as fast and straight as possible by moving their fingers in a vertical direction. At the beginning of each trial, participants were asked to set their fingers, indicated as white dots, at the home position, displayed as the reference line on the monitor. Next, the participants maintained their fingers for 2 s, after which the finger positions disappeared, and a green target and cursor were presented. After the participants held their fingers for an additional 3 to 4 s, the color of the target turned magenta, which was the cue that participants should start the task. When the cursor was within the target, the reaching was judged successful. The next target in the sequence was not presented until participants successfully reached the current target or 5 s had elapsed since the current target was presented. Ten targets were presented either in random order (random trial) or in fixed order (fixed trial). The fixed trial was designed to eliminate the effect of differences in the order of target presentation on joint control of the virtual arm. Each trial consisted of ten point-to-point reaching movements. After a trial, participants returned their fingers to the home position. Ten blocks, each consisting of six trials, were performed with a 30 s rest between blocks (Fig. 1D). In summary, the experiment consisted of 10 reaching movements per trial, with each block comprising 6 trials. The participants completed a total of 10 blocks, resulting in a total of 600 reaching movements throughout the experiment.

### Data analysis

Offline analysis was performed in MATLAB (R2023a, The MathWorks Inc., Natick, MA, United States). First, the data were resampled at 50 Hz. We quantified task performance by the number of successful reaching movements, the directional error, and the movement time. An unsuccessful reach was defined as a reach in which the cursor did not reach the target within 5 s after the appearance of the target. The sum of successful reaches in a trial was calculated as the number of successful reaching movements. Thus, if participants did not reach any targets in a given trial, the number of successful reaching movements was 0. Next, the cursor trajectories were filtered by using a fourth-order low-pass Butterworth filter with a cut-off of 5 Hz, after which the data from each trial were divided into 10 reaching movements. The directional error was computed from the absolute perpendicular distance of the cursor from the straight line connecting two consecutive targets. The sum of the distances was subsequently divided by the number of timeframes during a reach. The mean of these values over ten reaches was defined as the directional error in a trial. The movement time was the elapsed time from the appearance of a target until the cursor reached the target. If participants did not reach the target, the movement time was 5 s. The mean movement time between two consecutive targets was calculated as the movement time in a trial. We then focused the change in the ratio of the amount of use of each joint during learning. To quantify the relative use of each joint, the ratio of the amount of joint angular change during a trial among the three joints was calculated. When participants used three joints equally, the relative use of each joint was 33.3%. To assess the ability to move the cursor effortlessly, we quantified the amount of angular change in the joint of the virtual arm. Specifically, the absolute value of the difference in joint angles between two consecutive time points in a trial was calculated and summed over a trial. This value was then normalized by the displacement of the cursor in the trial.

### Statistical analysis

We confirmed the normality of all the tested data by using Shapiro‒Wilk tests (*p* > 0.05). We performed paired *t* tests to compare task performance (number of successful reaching movements, directional error, and movement time) between the first block and the last block. In addition, a paired *t* test was conducted to test the differences in the amount of joint angular change summed across all joints of the virtual arm between the first and last blocks. The differences in the amount of joint angular changes among joints of the virtual arm and between the first and the last block were tested by using two-way repeated-measures analysis of variance (ANOVA). Two-way repeated-measures ANOVA was conducted to compare the relative use among joints of the virtual arm and between the first and the last block. Significant ANOVA results were followed up by post hoc multiple comparisons with Tukey’s method. The associations between the relative use of joints in the virtual arm and the number of successful reaching were assessed by Pearson’s correlation coefficients. The *p* values of the correlation analysis were corrected using the Holm-Bonferroni correction. All statistical analyses were performed using JASP version 0.18.3^42^ with a significance level of 0.05. The data were presented as mean ± SD.

### Supplementary Information

Supplementary Information.

## Data Availability

The datasets analyzed during the current study are available from the corresponding author upon reasonable request.
