# Supplementary material for: Motor learning in multijoint virtual arm movements with novel kinematics
Source: Sci Rep. 2024 May 7;14:10421. doi: 10.1038/s41598-024-60844-7 (PMC11074153; doi:10.1038/s41598-024-60844-7)
Supplement: Supplementary file 1 — Supplementary Information. [file 41598_2024_60844_MOESM1_ESM.docx]

**Supplementary Information**

***Title:* Motor learning in multijoint virtual arm movements with novel kinematics**

***Authors:*** Nagisa Inubashiri^1^, Shota Hagio^2,3^, and Motoki Kouzaki^1,3^

***Author Affiliations:***

^1^ Laboratory of Neurophysiology, Graduate School of Human and Environmental Studies, Kyoto University, Kyoto, Japan

^2^ Laboratory of Motor Control and Learning, Graduate School of Human and Environmental Studies, Kyoto University, Kyoto, Japan

^3^ Unit of Synergetic Studies for Space, Kyoto University, Kyoto, Japan

**Supplementary Methods**

**Simulation**

In order to examine the optimal usage of the virtual arm, we simulated the generation of desired trajectories for the virtual hand while minimizing the joint angular changes of the virtual arm and the error of the endpoint. We conducted the simulation using the Jacobian matrix which describes the relationship between joint angles and endpoint displacement of the virtual arm^43^. Based on the forward-kinematics model of the virtual arm, the Jacobian matrix (*J*) was computed as

$$\begin{aligned} J\left( \theta\right)=\left[ \begin{matrix} \frac{\partial x}{\partial\theta_{1}} & \frac{\partial x}{\partial\theta_{2}} & \frac{\partial x}{\partial\theta_{3}} \\ \frac{\partial y}{\partial\theta_{1}} & \frac{\partial y}{\partial\theta_{2}} & \frac{\partial y}{\partial\theta_{3}} \end{matrix} \right]\#\left( 1 \right) \end{aligned}$$

where $\theta$ represents the joint angles of the virtual arm. Also, *x* and *y* corresponds to the endpoint of the virtual arm. In this simulation, the trajectory of the virtual hand was assumed to be a straight line toward the target since participants were instructed to reach straight for the targets. Also, the step size was set as the distance from the starting position to the target divided by 0.1.

Joint angles of the virtual arm at each step were computed from

$$\begin{aligned} \dot{\theta}= J^{-1}\dot{P}\#\left( 2 \right) \end{aligned}$$

where *P* represents the endpoint (x,y) of the virtual arm. Since the Jacobian is not square because of the redundancy of the virtual arm kinematics, we used the pseudo-inverse of the Jacobian ($J^{-1}$). We simulated the trials in the last block (five random trials and one fixed trial) performed by each subject. We then calculated the relative use of the virtual joints. To be consistent with the experimental data, we then averaged the 6 trials experienced by each participant and made this value representative of the protocol experienced by each participant. One-way ANOVA was conducted to compare the relative use of the virtual joints in the simulation data.

**Reference**

1. Meredith, M. & Maddock, S. Using a Half-Jacobian for Real-Time Inverse Kinematics. *Proceedings of the International Conference on Computer Games: Artificial Intelligence, Design and Education*. (2004).

**
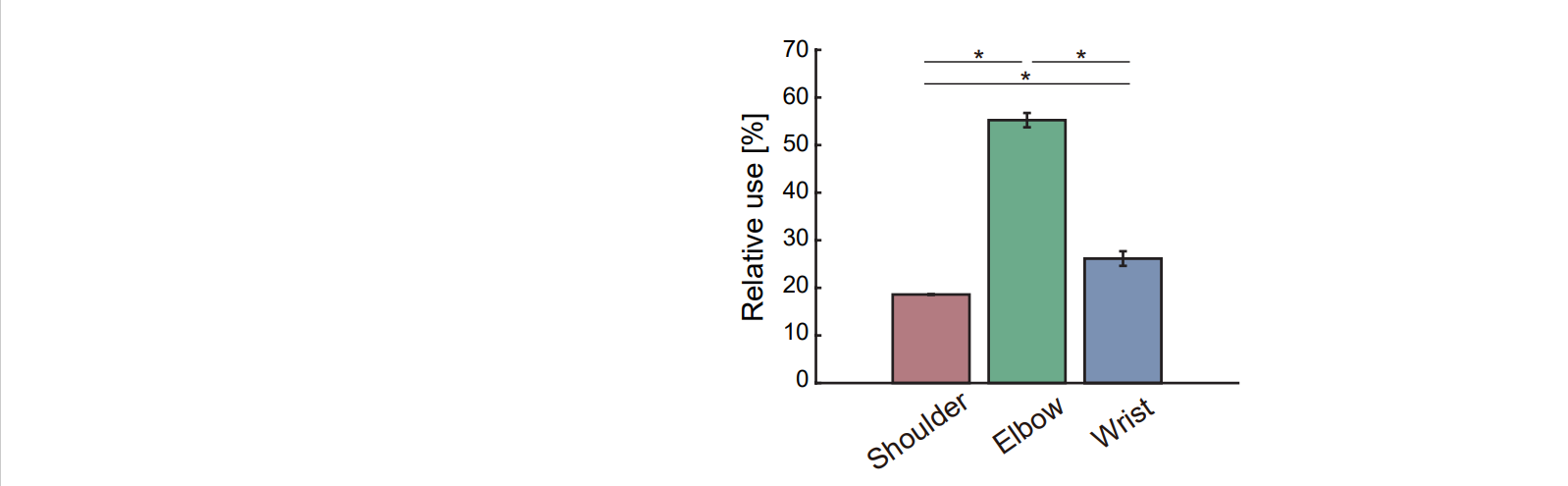
Supplementary Figures**

**Supplementary Figure S1. Relative use of the virtual joints calculated from simulation data.**

All trials in the last block for all participants were simulated. Relative use of the virtual shoulder, virtual elbow, and virtual wrist averaged across the representative values of the protocol experienced by each participant. The error bar indicates the SEM across the representative values of the protocol experienced by each participant. One-way ANOVA revealed a significant difference in relative use between virtual joints (shoulder, 18.6 ± 0.3%; elbow, 55.2 ± 6.6%; wrist, 26.2 ± 6.7%; *F*_2,54_ = 242.78, *p* < 0.001). *Post hoc* tests showed that the relative use of the virtual elbow was significantly greater than that of the other virtual joints (p < 0.001). * indicates *p* < 0.001.

**
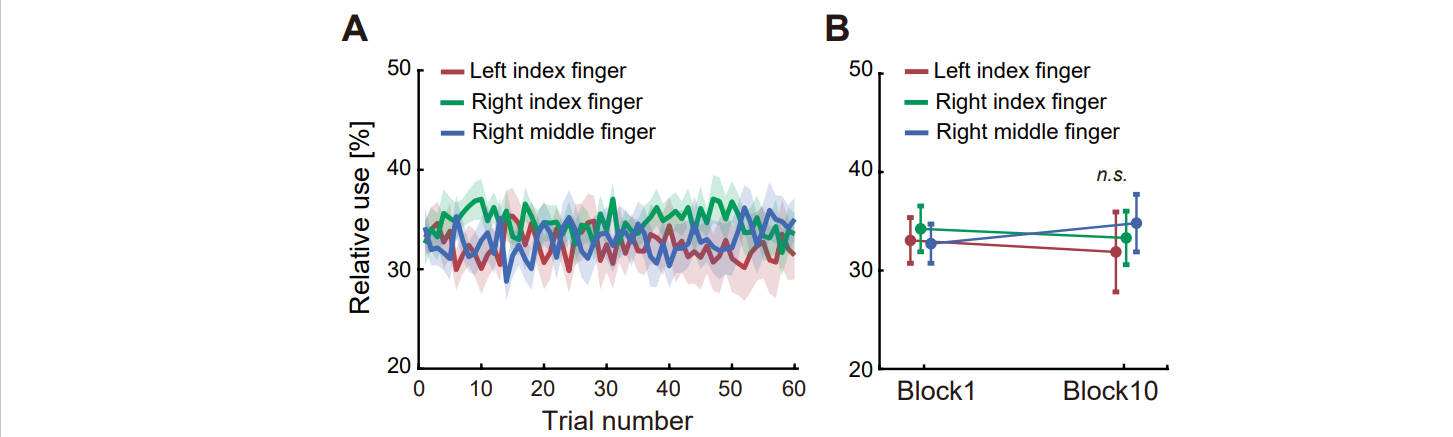
**

**Supplementary Figure S2. Relative use of each finger.**

(**A**) Relative use of the left index, right index, and right middle fingers averaged across participants. The red, green, and blue lines indicate the left index, the right index, and the right middle fingers, respectively. The shaded area represents the SEM. (**B**) Relative use of each finger in the first (trial number: 1-6) and last (trial number: 55-60) block. All error bars represent the SEM.
